# Childhood Maltreatment Influences Mental Symptoms: The Mediating Roles of Emotional Intelligence and Social Support

**DOI:** 10.3389/fpsyt.2019.00415

**Published:** 2019-06-21

**Authors:** Jiaxu Zhao, Xin Peng, Xiaomei Chao, Yanhui Xiang

**Affiliations:** ^1^Department of Psychology and Cognition and Human Behavior Key Laboratory of Hunan Province, Hunan Normal University, Changsha, China; ^2^Department of Preschool Education, Hunan Normal University, Changsha, China

**Keywords:** childhood maltreatment, SCL-90, emotional intelligence, social support, structural equation model

## Abstract

Childhood maltreatment and its influence on mental health are key concerns around the world. Previous studies have found that childhood maltreatment is a positive predictor of mental symptoms, but few studies have been done to explore the specific mediating mechanisms between these two variables. Previous studies have found that there is a negative correlation between childhood maltreatment and emotional intelligence and between childhood maltreatment and social support, both of which are strong indicators of mental symptoms. Therefore, in this study, we took emotional intelligence and social support as mediating variables, exploring their mediating effects between childhood maltreatment and mental symptoms via the structural equation modeling method. We recruited 811 Chinese college students to complete the Childhood Trauma Questionnaire (CTQ), the Symptom Checklist 90 Scale (SCL-90), the Wong Law Emotional Intelligence Scale (WLEIS), and the Perceived Social Support Scale (PSSS). The results showed a significant and positive correlation between childhood maltreatment and mental symptoms (*β* = 0.26, *P* < 0.001); meanwhile, social support played a significant mediating role in the influence of childhood maltreatment on emotional intelligence [95% confidence intervals, (−0.594 to −0.327)]; and emotional intelligence likewise played a significant mediating role in the effect of social support on mental symptoms [95% confidence intervals, (−0.224 to −0.105)]. These results indicated that childhood maltreatment not only directly increases the likelihood of developing mental symptoms, but also affects emotional intelligence through influencing social support and then indirectly increasing the likelihood of developing mental symptoms. This study provided a theoretical basis for ameliorating adverse effects of childhood maltreatment on mental symptoms by enhancing emotional intelligence and social support.

## Introduction

Previous researchers have explored the relationship between childhood maltreatment and mental symptoms, consistently finding that childhood maltreatment has a positive correlation with mental symptoms (1, 2). However, few studies have been done to further examine the specific mediating mechanisms between these two variables, and specifically, the potential for operable mediating variables to mitigate the adverse effects of childhood maltreatment on mental symptoms. Additionally, previous studies have shown that childhood maltreatment negatively affects emotional intelligence (3), and emotional intelligence can inhibit the emergence of mental symptoms (4). Other studies have found that childhood maltreatment negatively affects social support (5), and social support can also inhibit mental symptoms (6). Therefore, to expand on existing research, this study will use the structural equation modeling method to explore the mediating roles of emotional intelligence and social support in the relationship between childhood maltreatment and mental symptoms.

Childhood maltreatment refers to violent or other abusive actions by parents or other caregivers, causing physical and mental harm (7). It includes five aspects: emotional neglect, emotional abuse, physical neglect, physical abuse, and sexual abuse (8). Previous studies have shown that childhood maltreatment can damage social emotions, such as by making them more irritable, anxious, and depressed (9, 10). In addition, childhood maltreatment can also affect interpersonal relationships (11, 12), even leading to debilitating mental symptoms, such as post-traumatic stress disorder (PTSD) (13). Many studies have found a significant and positive correlation between childhood maltreatment and mental symptoms (14–16); this study further explores the specific mediating mechanisms between these two variables by using operable variables as mediating variables.

Previous studies have found that childhood maltreatment can negatively affect social emotions (17), as well as the ability to assess, regulate, and appropriately use emotions, which may lead to a negative impact on emotional intelligence (18, 19). Emotional intelligence is the ability of people to monitor the emotions of themselves and other people, guiding their thoughts and behaviors by discriminating and using information about emotions (20). Prior studies have shown that higher emotional intelligence can relieve stress and anxiety (21) and can improve people’s overall sense of life satisfaction (22). Furthermore, many studies have found that emotional intelligence has a significant and positive effect on inhibiting mental symptoms (23, 24). Therefore, we can hypothesize that emotional intelligence plays a mediating role in the effects of childhood maltreatment on mental symptoms.

Studies have also found that childhood maltreatment can lead to social withdrawal and behavior issues and bad social interaction behaviors (25–27). Punamäki et al. have further found that childhood maltreatment can negatively affect social support (28). Social support refers to the degree that people believe themselves to be cared for, loved, and respected in social networks (29). Previous studies have shown that higher social support can not only inhibit the development of depression, envy, and other negative emotions (30, 31) but can also enhance overall well-being as perceived by people (32). In addition, prior studies have further pointed out that social support negatively correlates with the occurrence of mental symptoms (33, 34). Some studies have demonstrated that social support can be a mediation of childhood maltreatment and psychological problems (e.g., current psychological adjustment, depression) (35, 36). Therefore, we hypothesize that social support also plays a mediating role in the effects of childhood maltreatment on mental symptoms. In addition, social support has been shown to positively affect the ability to regulate emotions, specifically by inhibiting people from generating negative emotions (37, 38). Furthermore, many studies have shown that social support can improve emotional intelligence, thus enhancing subjective well-being as perceived by people (39, 40). Therefore, we further hypothesize that childhood maltreatment may affect emotional intelligence by influencing social support, thus affecting mental symptoms indirectly in addition to directly.

In conclusion, the present study aimed to explore the mediating roles of emotional intelligence and social support in the relationship between childhood maltreatment and mental symptoms. Based on previous studies, we proposed the following two hypotheses: 1) Emotional intelligence and social support both play significant roles in mediating the relationship between childhood maltreatment and mental symptoms; 2) Childhood maltreatment also has an effect on emotional intelligence by influencing social support, thus indirectly increasing the likelihood of developing mental symptoms.

## Materials and Methods

### Participants

We used cluster sampling method and element sampling method to randomly select 811 participants from several universities in mainland China, including 217 males and 594 females. The ages of these participants ranged from 17 to 26 years old, an average age of 19.54 ± 1.86. The present study was approved by the Academic Committee of the School of Psychology of Hunan Normal University. All participants provided written informed consent before completing the questionnaires and were paid after completion.

### Measures

The questionnaires consist of five parts: The investigation of basic demographics, the Childhood Trauma Questionnaire (CTQ), the Symptom Checklist 90 Scale (SCL-90), the Wong Law Emotional Intelligence Scale (WLEIS), and the Perceived Social Support Scale (PSSS).

#### Childhood Trauma Questionnaire (CTQ)

The CTQ was authorized by Bernstein et al. (8). This scale consists of 28 items, including five subscales: emotional neglect, emotional abuse, physical neglect, physical abuse, and sexual abuse. The CTQ is scored on a five-point Likert-type scale, with higher scores indicating a stronger degree of childhood maltreatment. We used the adaptation of Zhao et al. (41) to evaluate the childhood maltreatment of our participants in this study. There are 23 items in this adaptation, and the sexual abuse subscales were eliminated due to cultural difficulties in obtaining accurate responses from participants. This adapted version has been proved to have high reliability and validity in the Chinese population (41). In this present study, the Cronbach’s alpha coefficient for the total scale was 0.64, and the Cronbach’s alpha coefficient for each of the subscales was as follows: emotional neglect: 0.78, emotional abuse: 0.75, physical neglect: 0.70, and physical abuse: 0.80.

#### Symptom Checklist 90 Scale (SCL-90)

The SCL-90 was authorized by Derogatis et al. (42). The scale consists of 90 items and is divided into 10 dimensions: Somatization, Obsessive–Compulsive, Interpersonal Sensitivity, Depression, Anxiety, Hostility, Phobic Anxiety, Paranoid Ideation, Psychoticism, and Others. It is scored on a five-point Likert-type scale, with higher scores indicating increased severity of mental symptoms. To evaluate the mental symptoms of our participants in this study, we used the adaptation of Tang and Cheng (43), which has been proven to have high reliability and validity in the Chinese population. In the present study, the Cronbach’s alpha coefficient for this scale was 0.96.

#### Wong Law Emotional Intelligence Scale (WLEIS)

The WLEIS was authorized by Wong and Law (44). The scale consists of 16 items and is divided into four subscales, including self-emotional assessment (SEA), others’ emotional assessment (OEA), regulations of emotions (ROE), and use of emotions (UOE). It is scored on a seven-point Likert-type scale, with higher scores indicating greater emotional intelligence. We used the adaptation of Bao et al. (45) to evaluate the emotional intelligence of our participants in this study, this adaptation has been shown to be highly reliable and valid among the Chinese population. In the present study, the Cronbach’s alpha coefficient for this scale was 0.90, and the Cronbach’s alpha coefficient for each of the subscales was as follows: SEA: 0.80, OEA: 0.82, ROE: 0.86, and UOE: 0.82.

#### Perceived Social Support Scale (PSSS)

The PSSS was authorized by Zimet et al. (46). The scale consists of 12 items and is divided into three dimensions, including family support, friends’ support, and others’ support. It is scored on a seven-point Likert-type scale, with higher scores indicating higher perception of social support. We used the adaptation of Kong et al. (47) to evaluate the social support of our participants in this study, which has been proven to be highly reliable and valid among the Chinese population. In the present study, the Cronbach’s alpha coefficient for this scale was 0.90.

### Data Analysis

We used AMOS 22.0 to evaluate the measurement model we built and test whether our indicators could reliably predict the latent variables well. Using an item-to-construct balance approach (48), we separated the four subscales of CTQ, the 10 dimensions of SCL-90, the 4 subscales of WLEIS, and the 3 dimensions of PSSS, to serve as indicators of the factors. If the measurement model fits well, we were to build the structural model, using the chi-square statistic, standardized root-mean-square residual (SRMR, 0.080 or less), root-mean-square error of approximation (RMSEA, 0.080 or less), and comparative fit index (CFI, 0.900 or more) as the indicators to test the model’s accuracy (49). At the same time, we used the Akaike Information Criterion (AIC) as an indicator to compare models, with lower AIC values indicating better fit (50). At last, we used the expected cross-validation index (ECVI) as an indicator to evaluate the potential for replication of the models, with lower ECVI values indicating greater potential for replication (51).

## Result

### Measurement Model

The latent variables in the measurement model included childhood maltreatment, emotional intelligence, social support, and SCL-90. The results showed that the data fitted well with the measurement model [χ^2^_(183,_ *_N_*_ = 811)_ = 1141.547, *P* < 0.001; RMSEA = 0.076; SRMR = 0.053; CFI = 0.920]. In addition, the latent variables correlated significantly with the factors that they are loading (*P* < 0.001). This indicated that the latent variables could well represent the observed variables and that all the latent variables were significantly correlated. The means (M), standard deviations (SD), and bivariate correlations among childhood maltreatment, emotional intelligence, social support, and mental symptoms are shown in **Table 1**.

**Table 1:** Descriptive statistics and bivariate correlations for all measures.

	M	SD	1	2	3	4	5
1. Age	19.54	1.86	1.000				
2. Childhood maltreatment	31.43	8.10	.003	1.000			
3. Emotional intelligence	80.95	12.51	.060	−.198**	1.000		
4. Social support	62.57	11.05	−.019	−.406**	.386**	1.000	
5. Mental symptoms	52.67	39.70	−.080*	.291**	−.283**	−.261**	1.000

*P < 0.05. **P < 0.01. ***P < 0.001

### The Evaluation of Rationality in Structural Model

**Figure 1:**
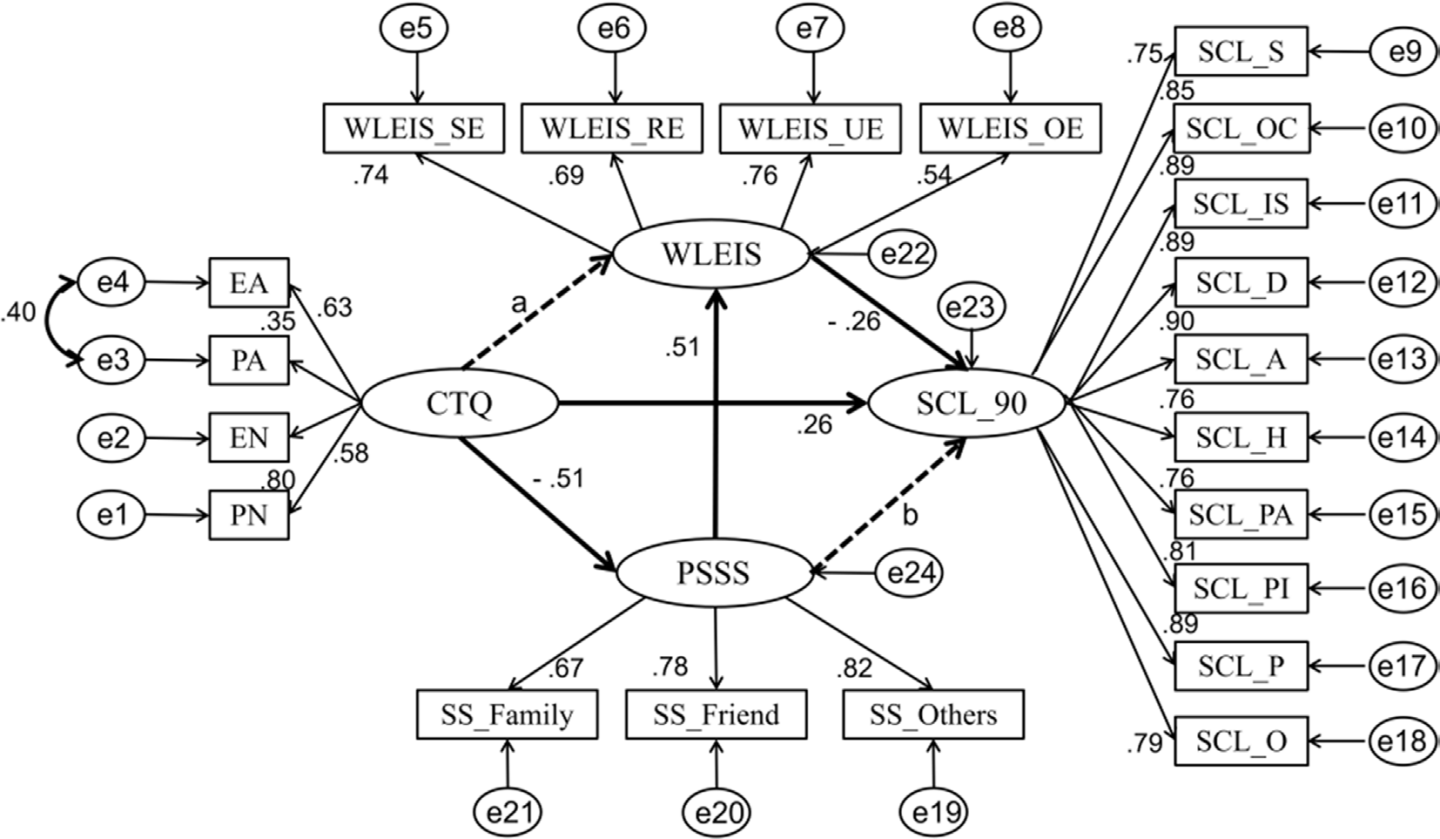
The mediating model factor loadings are standardized. Emotional abuse (EA), physical abuse (PA), emotional neglect (EN), and physical neglect (PN) are four subscales of the Childhood Trauma Questionnaire (CTQ); self-emotional assessment (WLEIS_SE), others’ emotional evaluation (WLEIS_OE), regulations of emotions (WLEIS_RE), and use of emotions (WLEIS_UE) are four subscales of the Wong Law Emotional Intelligence Scale (WLEIS); family’s support (SS_Family), friends’ support (SS_Friend), and others’ support (SS_Others) are three dimensions of the Perceived Social Support Scale (PSSS); Somatization (SCL_S), Obsessive–Compulsive (SCL_OC), Interpersonal Sensitivity (SCL_IS), Depression (SCL_D), Anxiety (SCL_A), Hostility (SCL_H), Phobic Anxiety (SCL_PA), Paranoid Ideation (SCL_PI), Psychoticism (SCL_P), and Others (SCL_O) are 10 dimensions of the Multi-Dimensional Scale of Symptom Checklist 90 Scale (SCL-90).

With the absence of mediating variables (emotional intelligence and social support), childhood maltreatment is significantly and positively related to mental symptoms (*β* = 0.26, *P* < 0.001) (criterion). We established in Model 1 that childhood maltreatment not only can directly affect mental symptoms but also can indirectly affect it by influencing emotional intelligence through social support. The results showed the fit of Model 1 to be accurate [χ^2^_(183,_ *_N_*_ = 811)_ = 1,041.547, *P* < 0.001; RMSEA = 0.076; SRMR = 0.053; CFI = 0.920], but the standardized path coefficients of Childhood maltreatment → Emotional intelligence (a, *β* = 0.55, *P* = 0.546) and Social support → Mental symptoms (b, *β* = 0.27, *P* = 0.274) were not significant. Therefore, we limited the coefficients of these two paths to zero when constructing Model 2, which includes complete mediating relationships. The results showed the fit of Model 2 to likewise be accurate [χ^2^_(185,_ *_N_*_ = 811)_ = 1,042.995, *P* < 0.001; RMSEA = 0.076; SRMR = 0.053; CFI = 0.920]. In addition, we found a statistically significant correlation between the error items of emotional abuse (EA) (e4) and physical abuse (PA) (e3) in childhood maltreatment (CTQ). Therefore, based on Model 2, we established Model 3. The results showed that Model 3 has a better fit on observed variables [χ^2^_(184,_ *_N_*_ = 811)_ = 946.305, *P* < 0.001; RMSEA = 0.072; SRMR = 0.051; CFI = 0.929]. Compared to Model 2, we found that Model 3 had a smaller χ^2^ [Δχ^2^_(1,_ *_N_*_ = 811)_ = 96.690, *P* < 0.001] and a smaller AIC. This indicates that Model 3 is a better fit than Model 2 (see **Table 2**); hence, we chose to use Model 3 as the final structural model (see **Figure 1**).

**Table 2:** Fit indices among Model 1, Model 2, and Model 3.

	χ2/df	CFI	RMSEA	SRMR	AIC	ECVI
Model 1	5.691	0.920	0.076	0.053	1137.547	1.404
Model 2	5.638	0.920	0.076	0.053	1134.995	1.401
Model 3	5.143	0.929	0.072	0.051	1040.305	1.284

RMSEA, root-mean-square error of approximation; SRMR, standardized root-mean-square residual; CFI, comparative fit index; AIC, Akaike information criterion; ECVI, expected cross-validation index.

### The Significance Test of Mediating Variables

Based on the above, we used the Bootstrap estimation procedure to explore the stability of the mediating effects. We randomly generated 2,000 bootstrap samples (*N* = 811) from the original data set by random sampling. The results showed that mediating variables play significant roles in 95% confidence intervals. As we show in **Table 3**, childhood maltreatment significantly influences emotional intelligence through social support [95% confidence intervals, (−0.594 to −0.327)], and social support has a significant effect on mental symptoms via emotional intelligence [95% confidence intervals, (−0.224 to −0.105)].

**Table 3:** Standardized indirect effects and 95% confidence intervals.

Pathways	Estimate	Lower	Upper
Childhood maltreatment → Social support → Emotional intelligence	−0.260	−0.594	−0.327
Social support → Emotional intelligence → Mental symptoms	−0.133	−0.224	−0.105

### Gender Difference

We used the four latent variables to test differences between genders. The results showed that there are no perceivable gender differences in childhood maltreatment [*t*_(811)_ = 0.858, *P* = 0.391], emotional intelligence [*t*_(811)_ = 0.736, *P* = 0.462], or mental symptoms [*t*_(811)_ = −0.805, *P* = 0.421], but that the gender differences in social support [*t*_(811)_ = −2.549, *P* = 0.011] is significant, with female participants scoring higher than male participants. Based on these results, we further investigated the stability of the gender differences we found in the structural model.

We used a multi-group analysis to determine whether the path coefficients have significant differences in the models between gender differences. Referring to the study of Byrne (52), we established two models on the basis of keeping the basic parameters (factor loadings, error variances, and structural covariances) stable. One allowed free estimations of the path coefficients between two genders (unconstrained structural paths), while the other limited them (constrained structural paths). The results showed significant differences between these two models [∆χ^2^_(25,_ *_N_*_ = 811)_ = 66.838, *P* < 0.001]. Meanwhile, when we compared other parameters in these two models, both models have good fits (see **Table 4**). Therefore, the parameter-limited deformable models in multiple groups are generally acceptable. In addition, because ∆χ^2^ is easily influenced by large sample sizes and then reaches a significant level, the critical ratio of standard deviation (CRD) was further calculated (53). According to the decision rules, the absolute value of CRD is greater than 1.96, meaning that the two parameters are significantly different at a significance level of *P* < 0.05. The results showed that there was no significant difference in the structural paths of all variables (CRD_CTQ → PSSS_ = 1.406, CRD_PSSS → WLEIS_ = −0.076, CRD_WLEIS → SCL-90_ = 1.285, and CRD_CTQ → SCL-90_ = 0.485). Therefore, we believed that there was no significant difference in the comparison between the two models and that there was no significant gender differences in the specific coefficients according to the value of CRD.

**Table 4:** Unconstrained and constrained structural paths across genders.

	χ^2^/df	CFI	RMSEA	SRMR	AIC	ECVI
Unconstrained SP	3.287	0.922	0.053	0.062	1402.754	1.734
Constrained SP	3.257	0.918	0.053	0.072	1416.571	1.757

RMSEA, root-mean-square error of approximation; SRMR, standardized root-mean-square residual; CFI, comparative fit index; AIC, Akaike information criterion; ECVI, expected cross-validation index.

## Discussion

The purpose of the present study was to explore mediating roles of emotional intelligence and social support in the relationship between childhood maltreatment and mental symptoms. The results showed that while emotional intelligence and social support both do not directly mediate the relationship between childhood maltreatment and mental symptoms, childhood maltreatment affects emotional intelligence by influencing social support, thus indirectly increasing the likelihood of developing mental symptoms.

According to the results of correlation analysis, there was a significant and positive correlation between childhood maltreatment and mental symptoms, which was also confirmed by the regression coefficients in the structure model. This is consistent with the results of previous studies. Lots of studies have shown that additional damaging consequences of childhood maltreatment, including cognition, emotions, and behaviors (e.g., lower self-compassion, more psychological distress, more suicide attempts), may increase the propensity of people toward developing mental symptoms (54–56). Furthermore, based on Model 3, we further tested the significance of mediating variables and found that there are two complete mediating relationships in the model: 1) Childhood maltreatment → Social support → Emotional intelligence; 2) Social support → Emotional intelligence → Mental symptoms. These results showed that childhood maltreatment negatively affects emotional intelligence by influencing social support, and social support affects the severity of mental symptoms by influencing emotional intelligence. That is, childhood maltreatment affects emotional intelligence by influencing social support, thus indirectly increasing the tendency of people to develop mental symptoms.

The results of the significance test on mediating variables are different from our hypothesis 1, but consistent with our hypothesis 2. We explained them specifically as follows: previous studies have shown that childhood maltreatment negatively affects cognitive, emotional, and social development, leading to interactive and communicative problems, and also making people more susceptible to have negative emotions in general and observable behavior problems, such as emotional maladjustment and restlessness, hyperactivity, antisocial behaviors, and delinquent behaviors (57). In addition, Schwartz found that childhood maltreatment had a significant negative effect on emotional intelligence (3). Other studies have also found that social support plays an important role in inhibiting and mitigating the long-term adverse consequences of childhood maltreatment, such as by inhibiting development of anxiety and depression, by enhancing the ability to self-regulate emotion (35, 37, 58), and by improving emotional intelligence (59). Therefore, we believed that social support plays a complete mediating role between childhood maltreatment and emotional intelligence. Furthermore, previous studies have clearly shown that social support can significantly and negatively affect mental symptoms (33, 34), but additional research was needed to explore the mediating mechanism in this relationship. However, some studies have found that social support can significantly and positively affect emotional intelligence (59). Many studies also have found that emotional intelligence can improve life satisfaction, reduce stress, and inhibit the development of mental illnesses, thus negatively affecting mental symptoms (4, 21). Therefore, we believed that emotional intelligence plays a complete mediating role between social support and mental symptoms.

Finally, we combined the results of the two complete mediating relationships: that is, the clue: Childhood maltreatment → Social support → Emotional intelligence → Mental symptoms. As a result, we see that childhood maltreatment affects emotional intelligence by influencing social support, which in turn influences the development of mental symptoms. This was also consistent with previous studies: childhood maltreatment lowers social support (27), which leads to lower emotional intelligence (56), resulting in the development of mental symptoms (24). Therefore, we believed that the effect of childhood maltreatment on mental symptoms is mediated by two successive causal mediators: social support and emotional intelligence.

The present study had some limitations. Firstly, although we have demonstrated the transgender stability of the model, the gender distribution in our sample was not well coordinated. Secondly, the results of our structural equation model have preliminarily explored the causal relationship between childhood maltreatment and mental symptoms, but additional longitudinal research is needed to assess its stability.

In conclusion, this present study demonstrated a positive correlation between childhood maltreatment and mental symptoms, verifying the mediating mechanisms in this relationship for the first time. That is, childhood maltreatment has an effect on emotional intelligence through its influence on social support, which affects the development and degree of mental symptoms. The study provided a theoretical basis for inhibiting mental symptoms through addressing their social support and emotional intelligence. It also significantly expands our understanding of childhood maltreatment, social support, emotional intelligence, and mental symptoms.

## Ethics Statement

The protocol was approved by the Academic Committee of the School of Psychology of Hunan Normal University.

## Author Contributions

JZ: Study design, data collection, data analysis, and paper revision. XP: Paper revision. XC: Paper revision. YX: Study design, data collection, data analysis, and paper revision.

## Funding

This work was supported by grants from the Nature Science Foundation of Hunan Province (NO. 2019JJ40195).

## Conflict of Interest Statement

The authors declare that the research was conducted in the absence of any commercial or financial relationships that could be construed as a potential conflict of interest.
